# Hemophagocytic lymphohistocytosis in trisomy 21: successful treatment with interferon inhibition

**DOI:** 10.1186/s12969-022-00764-w

**Published:** 2022-11-18

**Authors:** Allison Guild, Jordan Fritch, Sachit Patel, Adam Reinhardt, Melissa Acquazzino

**Affiliations:** 1 Department of Pediatrics, University of Nebraska Medical Center, Omaha, NE USA; 2 Department of Pediatrics, Division of Hematology Oncology, University of Nebraska Medical Center, Omaha, NE USA; 3 Department of Rheumatology, Boystown National Research Hospital, Omaha, NE USA

**Keywords:** Trisomy 21, Down syndrome, HLH, Interferonopathy, Interferon-gamma, IFNγ, Emapalumab, Baricitinib

## Abstract

**Background:**

Hemophagocytic lymphohistiocytosis (HLH) is a life-threatening condition of immune dysregulation primarily driven by the cytokine interferon gamma. It can be either a genetic or acquired disorder associated with infection, malignancy, and rheumatologic disorders. Trisomy 21 can express a wide range of phenotypes which include immune dysregulation and shares inherent pathophysiology with a group of disorders termed interferonopathies. Knowledge of this overlap in seemingly unrelated conditions could provide a basis for future research, and most importantly, alternative therapeutic interventions in acute life threatening clinical scenarios. Herein, we describe two patients with trisomy 21 presenting with HLH that was refractory to conventional treatment. Both patients were successfully managed with novel interventions targeting the interferon pathway.

**Case presentation:**

We describe a 17-month-old male and 15-month-old female with trisomy 21 presenting with a myriad of signs and symptoms including fever, rash, cytopenias, and hyperferritinemia, both ultimately diagnosed with HLH. Each had relapsing, refractory HLH over time requiring several admissions to the hospital receiving conventional high dose corticosteroids and interleukin-1 inhibition therapy. Successful steroid-free remission was achieved after targeting interferon inhibition with emapalumab induction followed by long-term maintenance on baricitinib.

**Conclusion:**

To our knowledge, these are the first reported cases of relapsed, refractory HLH in patients with trisomy 21 successfully treated with emapalumab and transitioned to a steroid-sparing regimen with oral baricitinib for maintenance therapy. Trisomy 21 autoimmunity and HLH are both thought to be driven by interferon gamma. Targeting therapy toward interferon signaling in both HLH and autoimmunity in trisomy 21 may have potential therapeutic benefits. Further investigation is needed to determine if trisomy 21 may predispose to the development of HLH given this common pathway.

## Background

Down syndrome is caused by trisomy 21, the occurrence of three copies of human chromosome 21. It is the most common chromosomal anomaly affecting approximately 1 in 800 newborns worldwide [1]. In addition to individuals with trisomy 21 having varying levels of intellectual disability, there are many associated co-morbidities including congenital heart disease, hearing loss, hematological malignancies, and immune dysregulatory conditions. Hypothyroidism, type I diabetes mellitus, celiac disease, and Down syndrome associated arthritis [2] occur at a disproportionately high rate when compared to healthy controls. It has been well established that trisomy 21 is associated with immune deficits, with both the innate and adaptive responses being affected [3]. Interferon gamma (IFNγ), a cytokine that is an important activator of macrophages, is found at elevated levels in children with trisomy 21 [4–6]. Other cytokines found downstream of interferon (IFN) signaling, including IL-6, IL-22, TNF⍺, and VEGF-A are also elevated in those with trisomy 21 [7]. This overexpression may be due to the fact that four of the six IFN receptor subunits are found on chromosome 21 [8]. The overexpression of these genes is thought to contribute to the development of immune dysregulation in trisomy 21 [8, 9]. This has prompted some to consider trisomy 21 in a group of disorders termed interferonopathies [10]. Further weight to this concept is supported by evidence that triplication of the IFN receptor in mouse models led to increased expression of all four IFN receptors and an exacerbated immune response, whereas normalization of IFN receptor gene dosage rescued multiple key phenotypic changes associated with trisomy 21 [11].

As IFNγ is an activator of macrophages, it has a central role in the pathogenesis of hemophagocytic lymphohistiocytosis (HLH), a life-threatening condition caused by overactivation of the immune system resulting in hypercytokinemia, hyperinflammation, and multi-organ failure. Diagnosis can be made if a patient meets five of the following eight criteria: fever > 38.3 C, splenomegaly, cytopenias, hyperferritinemia, hypertriglyceridemia and/or hypofibrinogenemia, low or absent natural killer (NK) cell activity, hemophagocytosis in bone marrow, spleen, liver, or lymph nodes, and elevated soluble CD25/soluble interleukin-2 receptor (sIL-2r) exceeding established values [12]. Classically, the diagnosis can be divided into primary (inherited) or secondary (acquired) disease. Primary HLH often presents in young children, has a clear genetic etiology, and has poor long-term prognosis without allogeneic hematopoietic stem cell transplantation [13, 14]. Secondary HLH is often the result of infection, malignancy, or in association with immune disease [14]. When associated with rheumatologic diseases, HLH is often referred to as macrophage activation syndrome (MAS) [14, 15]. The immune dysregulation in HLH is mainly thought to be driven by IFNγ [16]. Current standard of care treatment per protocol HLH-2004 aims to control the immune dysregulation with chemotherapy and steroids as a bridge to curative hematopoietic stem cell transplant [12, 17]. In those with recurrent HLH, often treatment ends with bone marrow transplant as well. In 2018, emapalumab (Gamifant), an IFNγ antibody, was FDA-approved for the treatment of pediatric and adult patients with primary HLH who have refractory, recurrent, or progressive disease or intolerance with conventional HLH therapy [18].

Additionally, janus kinase inhibition has also been proposed as a potential target in HLH treatment due to downstream impacts on IFN. Cells in individuals with trisomy 21 are hypersensitive to IFN stimulation, as demonstrated by increased janus kinase signal transducer and activator of transcription (JAK-STAT) signaling [19], making this a promising potential therapy.

Given the relationship of IFN in both HLH and trisomy 21, we targeted IFN suppression in two patients with trisomy 21 presenting with relapsed, refractory HLH/MAS.

## Case presentations

### Case 1

A 17-month-old male with trisomy 21, celiac disease, and history of complete atrioventricular septal defect status-post repair presented with persistent fevers, rash, hepatosplenomegaly, and serositis and arthritis on imaging. Labs revealed anemia (9.0 g/dL), thrombocytopenia (30,000), and elevated inflammatory markers, including ESR (81 mm/h), CRP (13.3 mg/dL), and ferritin (674 ng/ml). Initial investigation included a broad infectious workup, notably negative for CMV, EBV, adenovirus, parvovirus, hepatitis B, hepatitis C, and HIV. Immunological workup was largely unremarkable, with normal C3, C4, and IgG levels, along with normal NK cell function. IgA and IgM were mildly elevated at 225 mg/dL and 204 mg/dL, respectively. Initial cytokine testing included IL-6 only, which was elevated at 105 pg/mL. Hematological and oncological workup included bone marrow and inguinal lymph node biopsies which displayed no evidence of malignancy or HLH. Ultimately, a presumptive diagnosis of systemic juvenile idiopathic arthritis (sJIA) was made.

He therefore was promptly initiated on naproxen and a prednisolone bridge while beginning interleukin-1 (IL-1) inhibition via twice daily 2 mg/kg anakinra. He had several sJIA flares over the next several months manifested by recurrent fevers, rash, elevated inflammatory markers, and worsening arthralgias. Anakinra was discontinued and replaced with alternative IL-1 inhibition via monthly canakinumab. He also received maintenance prednisolone.

Despite the above therapies and dose adjustments, he continued to have frequent admissions for fevers and thrombocytopenia following infections. Finally, after an exhaustive infectious, malignant, and immune workup, the possibility of relapsing, refractory HLH/MAS was proposed. The diagnosis of HLH/MAS was ultimately made during an admission around 34 months of age and supported by persistent fever, hyperferritinemia (49,073 ng/mL), thrombocytopenia (59,000), anemia (8.4 g/dL), hypofibrinogenemia (50 mg/dL), elevated sIL-2r (13,079 U/ml), a highly specific marker for HLH [20], and hemophagocytosis visualized on repeat bone marrow biopsy. Supporting clinical criteria included transaminitis, hyponatremia, and associated coagulopathy. He was treated aggressively with high-dose methylprednisolone at 15 mg/kg twice daily for six total doses along with discontinuation of canakinumab and re-initiation of daily anakinra. The patient was discharged home on anakinra at 8 mg/kg daily and dexamethasone taper starting at 10 mg/m^2^/day over eight weeks per HLH-2004 protocol. Genetic testing for hereditary HLH, which included the four most common genes associated with familial HLH, PRF1, STX11, STXBP2, and UNC13D, along with several genes associated with X-linked lymphoproliferative disease and Griscelli syndrome, were all negative.

**Fig. 1:**
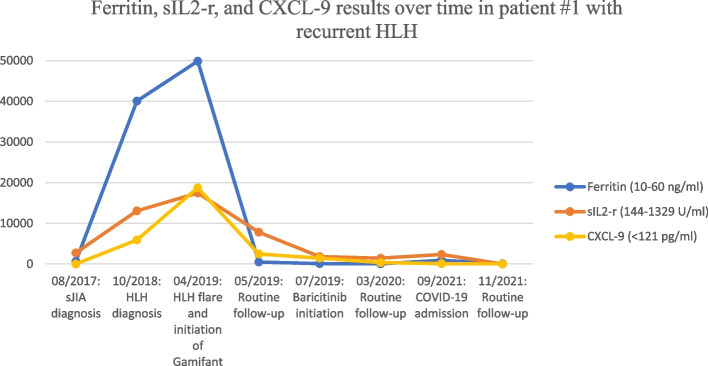
Various labs, including ferritin, soluble interleukin 2 receptor (sIL2-r), and chemokine ligand 9 (CXCL-9) values were trended over four years. The maximum values for a chosen time frame are included

After six months of treatment, he again suffered a relapsing course. Given ongoing episodes of macrophage activation despite high doses of IL-1 inhibition, extensive multi-disciplinary discussions led to approved use of emapalumab, given its known efficacy in primary HLH, in addition to ongoing anakinra and dexamethasone. Emapalumab was administered twice weekly for 10 weeks, starting with 1 mg/kg and titrated up to maximum of 3 mg/kg. Several inflammatory markers, including ferritin, sIL-2r, and chemokine ligand 9 (CXCL-9), a chemokine induced by IFNγ, were frequently monitored (see Fig. 1). While on emapalumab, the patient had both clinical and laboratory improvement. The patient was weaned off emapalumab and transitioned to twice daily baricitinib at 2 mg per dose (0.26 mg/kg/day). The decision to transition to baricitinib was largely due to cost efficacy and easier administration in that the patient could avoid long-term need for infusions. Baricitinib has been efficacious in treatment of interferonopathies given its suppression on IFNγ, so was trialed as maintenance in this patient, which ended up also being efficacious.

He did require re-hospitalization at 5 years of age for acute respiratory failure secondary to COVID-19 pneumonia. There was associated hyper-inflammation but the patient did not develop HLH/MAS at that time. He was maintained on baricitinib and anakinra throughout the illness. He did require a short course of corticosteroids for respiratory indications during hospitalization, which were able to be weaned off without relapse of hyper-inflammation or HLH/MAS. To date he continues to do well on maintenance anakinra and baricitinib without additional disease flare for over two years.

### Case 2

A 15-month-old female with trisomy 21, recurrent otitis media, persistent fevers and rash, and recurrent thrombocytopenia was admitted with fever, emesis, and acute liver failure. Initial infectious workup was negative for acute CMV, EBV, adenovirus, parvovirus, HSV, and HIV. Bone marrow biopsy was negative for malignancy and without evidence of hemophagocytosis. After an extensive infectious, malignant, and immune workup, she was diagnosed with a form of steroid-dependent HLH. Diagnosis of HLH was made based on fevers, hyperferritinemia (6,838 ng/ml), pancytopenia (WBC 1,900 though ANC > 1000, Hgb 6.2 g/dL, platelets 44,000), hypofibrinogenemia (96 mg/dl), elevated sIL-2r (12,390 U/ml), elevated sCD163 (8878 ng/mL) and decreased NK cell count and function with poor cytotoxicity, as evidenced by low CD107a, which can be used as a marker for NK cell activity. Additionally, she had decreased percentage of NK cells expressing perforin. Supporting clinical criteria for the diagnosis for HLH included transaminitis, vomiting, and weight loss. Her initial presentation was complicated by *Streptococcus pneumoniae* bacteremia and DIC. The patient was initiated on anakinra, along with an eight-week dexamethasone taper and etoposide, though delayed initially due to liver injury, per HLH-2004 protocol. Genetic testing for hereditary HLH, which included 14 associated genes, was negative. The etiology of her HLH was initially suspected to be secondary to an infectious trigger or underlying primary immunodeficiency given her low NK cell count and poor function. Of note, on initial presentation, she had normal immunoglobulin levels and normal pneumococcal serologies, suggesting functional B cells.

**Fig. 2:**
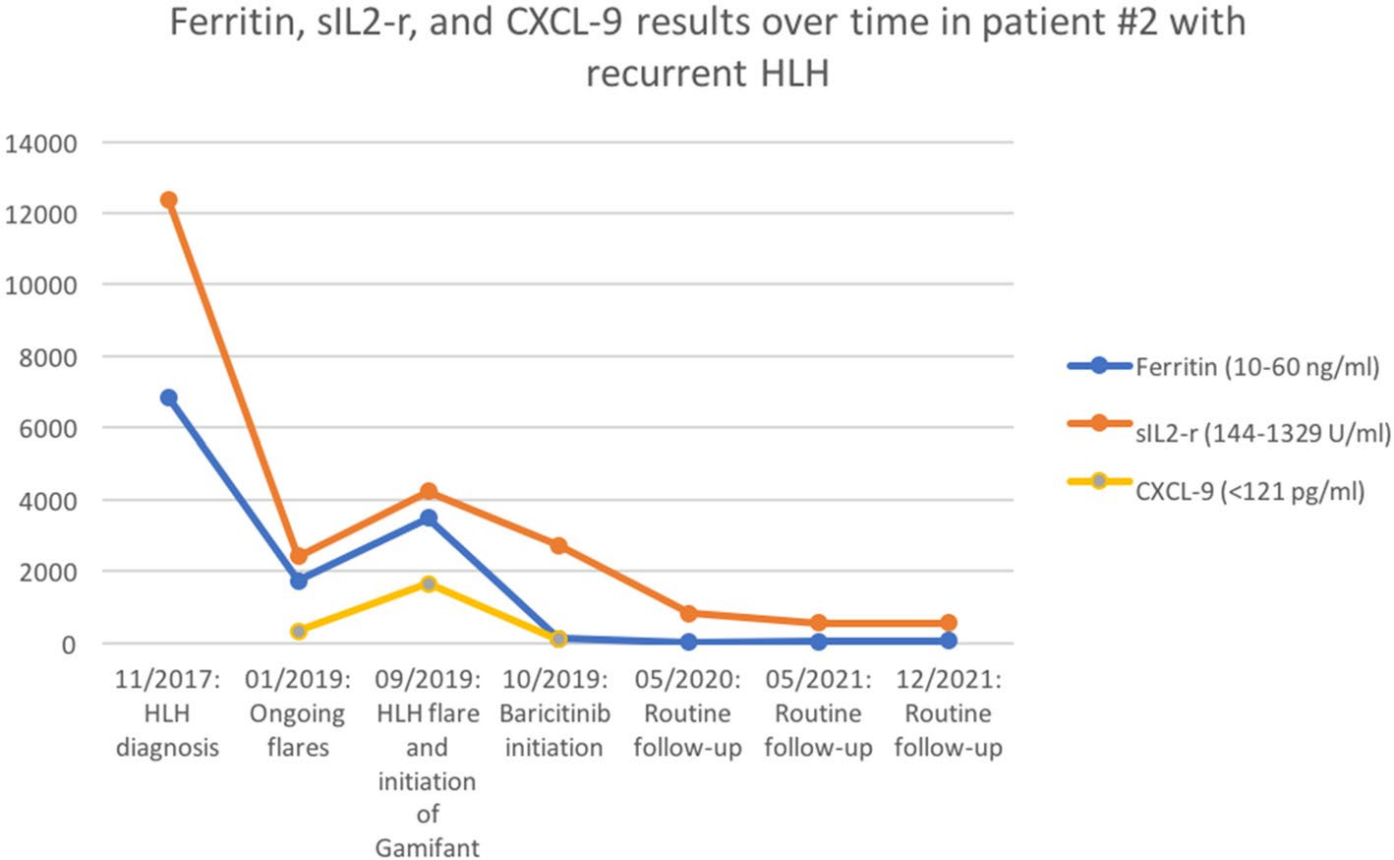
Ferritin, soluble interleukin-2 receptor (sIL2-r), and chemokine ligand 9 (CXCL-9) values were trended over four years. The maximum values for a chosen time frame are included on above graph

By three years of age, she continued to have repeated flares of macrophage activation, mainly triggered by attempts at weaning steroids as well as frequent infections, including viral sources, MRSE bacteremia, *Clostridium difficile* colitis, abdominal abscess, and cellulitis. Given her frequent exacerbations with steroid-dependence and despite high doses of IL-1 inhibition with both anakinra and canakinumab trials, she was initiated on emapalumab at 1 mg/kg twice weekly. Emapalumab was chosen given its efficacy in primary HLH. It was proposed that her HLH was due to underlying immune dysregulation and hyper-interferon signaling associated with trisomy 21. A baseline CXCL-9 was elevated at 303 pg/ml, affirming increased IFNγ signaling. While on emapalumab, ferritin, sIL-2r, CXCL-9, and other inflammatory markers were frequently monitored (see Fig. 2). She had both clinical and laboratory improvement so twice daily baricitinib was initiated at 2 mg per dose (0.3 mg/kg/day) after about five weeks of emapalumab treatment. Continued stability allowed successful wean of emapalumab after ten total weeks of treatment. She has continued to do well without return of disease for three years on maintenance baricitinib, allowing successful withdrawal of corticosteroids for the first time since diagnosis, and an ongoing slow wean of anakinra.

## Discussion

Those with trisomy 21 are highly predisposed to multi-organ immune dysregulation, likely due in part to hyperactivation of IFN signaling [9, 10, 21]. A recently described group of auto-inflammatory disorders characterized by dysregulation in IFN signaling are called interferonopathies [22]. These monogeneic disorders, some of which include Aicardi-Goutières syndrome (AGS), monogenetic forms of lupus, chronic atypical neutrophilic dermatosis with lipodystrophy and elevated temperatures (CANDLE), stimulator of IFN genes (STING)-associated vasculopathy with onset in infancy (SAVI), are caused by a mutation leading to upregulation of type I IFN, which ultimately is involved in the pathogenesis of the diseases [23]. Given trisomy 21 has elevated IFN levels [4–6], its proteome has multiple ties to IFN signaling [7], and many of the changes are similar to those observed in type I interferonopathies [10], it is reasonable to think of trisomy 21 similarly to an interferonopathy [8, 9, 11]. It has often been classified as a ‘mild interferonopathy,’ as the interferon activation is milder than that in patients with monogeneic interferonopathies [24]. Patients with trisomy 21 are likely more susceptible to developing HLH, especially following or during viral illnesses as the additional circulating IFN may reach the necessary threshold to tip them over into recurrent HLH, though literature regarding this potential association is scarce. Case reports include a 10-year-old female with trisomy 21 who developed HLH as a complication of *M. pneumoniae *infection [25]. Authors argued that the patient’s underlying immune dysregulation secondary to trisomy 21 most likely led to more severe infection, and ultimately HLH. Another case report describes a 3-year-old male with trisomy 21 in whom a severe SARS-CoV-2 infection triggered secondary HLH [26]. The underlying immune dysregulation in trisomy 21 patients, including known NK cell dysfunction [27–29], likely does contribute to their development of HLH, as this leads to decreased ability to clear infections. As seen in the second patient case we described above, her NK cell dysfunction likely contributed to her frequent HLH/MAS flares associated with infections. Regardless, with the elevated baseline IFN activation in those with trisomy 21, it is reasonable that early IFN inhibition may prevent or provide rescue for these patients regarding elevated HLH risk.

A mouse model with overexpression of IFN receptor genes, to mimic trisomy 21, showed that JAK 1/2 inhibitors provide therapeutic benefits such as blocked lethal immune responses and reduced cytokine production [30]. As HLH is also a disease of IFN over-activation, mouse models with IFNγ-blocking antibodies induced recovery and improved survival in two separate murine models [31]. Other murine models suggest that inhibiting multiple cytokine signaling pathways, through JAK inhibitors for example, may be more efficacious in the treatment of HLH compared to targeting IFNγ alone [32]. In preclinical murine models, treatment with ruxolitinib, a JAK 1/2 inhibitor, has been reported to improve inflammatory pathologies such as weight loss, organomegaly, and cytopenias, as well as survival [33].

With emapalumab’s FDA-approval for use in relapsing, refractory primary HLH in 2018, many patients have been effectively treated, and able to proceed to hematopoietic stem cell transplant [34]. Its use in secondary HLH, however, remains limited. A group of 14 pediatric patients with HLH/MAS secondary to sJIA all had resolution of their disease after treatment with emapalumab [35]. Case reports detail successful use in a woman with HLH/MAS secondary to adult onset Still’s disease [36] and resolution of EBV-associated HLH without an HLH-gene mutation in a 20-month old [37], similar to our described pediatric patients. Currently, there is an ongoing clinical trial evaluating the efficacy and safety of emapalumab in children and adults with secondary HLH/MAS [38]. In terms of oral JAK inhibitor use, its future use in HLH is promising. In the pediatric population, Zhang et al [39] detailed safe and efficacious use of ruxolitinib as first-line therapy in 12 children with secondary HLH, in which the overall response rate at end of 28-days of treatment was 83.3%. This allowed the patients to avoid the toxic effects secondary to standard chemotherapy treatment regimens. There are case reports, mainly in adult patients [40–42], of ruxolitinib used in refractory HLH salvage therapy with favorable results. Ruxolitinib was also used successfully as salvage treatment in an 11-year-old with refractory HLH [43]. It appears there is no current literature discussing specific use of baricitinib in HLH/MAS.

Management of other interferonopathies has found success in targeting the interferon response. Therapy targeted at inhibiting JAK has shown both clinical and laboratory improvement in patients with the monogeneic interferonopathies CANDLE and SAVI [44]. In trisomy 21 specifically, two cases have been reported of successful JAK inhibition with tofacitinib in patients with autoimmune alopecia areata [45]. These two patients are part of a larger cohort enrolled in the Crnic Institute Human Trisome Project’s clinical trial, which is evaluating tofacitinib’s safety and efficacy in treatment of immune skin conditions in trisomy 21 [46]. Literature regarding emapalumab use in patients with trisomy 21 is lacking, however.

To our knowledge, the two cases above are the first reported cases of relapsed, refractory HLH/MAS in patients with trisomy 21 successfully induced with emapalumab, an IFNγ antibody, and transitioned to a steroid sparing regimen with oral baricitinib, a JAK inhibitor, for maintenance interferon suppression. Early use of IFN suppression in this population can help abort HLH flares while chronic suppression helps prevent further flares.

## Conclusion

We report these cases to show that inhibition of interferon pathway through IFNγ inhibition and long term suppression with JAK inhibitors have potential therapeutic benefits in those with HLH and trisomy 21, as well as other disorders driven by interferon, which could lead to more targeted and earlier therapies. Further investigation is also needed to determine if trisomy 21 may predispose to the development of HLH given this common pathway.

## Data Availability

Not applicable.

## Abbreviations

AGS: Aicardi-Goutières syndrome
CANDLE: Chronic atypical neutrophilic dermatosis with lipodystrophy and elevated temperatures
CMV: Cytomegalovirus
CXCL-9: Chemokine ligand 9
EBV: Epstein-Barr virus
HIV: Human immunodeficiency virus
HLH: Hemophagocytic lymphohistiocytosis
HSV: Herpes simplex virus
IFN: Interferon
IFNγ: Interferon-gamma
IL: Interleukin
JAK-STAT: Janus kinase-signal transducer and activator of transcription
MAS: Macrophage activation syndrome
NK: Natural killer
SAVI: Stimulator of IFN genes (STING)-associated vasculopathy with onset in infancy
sIL2-r: Soluble interleukin-2 receptor
sJIA: Systemic juvenile idiopathic arthritis
STING: Stimulator of IFN genes
TKI: Tyrosine kinase inhibitor
